# Glucosamine suppresses proliferation of human prostate carcinoma DU145 cells through inhibition of STAT3 signaling

**DOI:** 10.1186/1475-2867-9-25

**Published:** 2009-09-10

**Authors:** Viktor Chesnokov, Chao Sun, Keiichi Itakura

**Affiliations:** 1 Department of Molecular Biology, Beckman Research Institute of the City of Hope, 1450 East Duarte road, Duarte, California 91010, USA

## Abstract

**Background:**

Glucosamine is known as a toxic agent for several malignant cell lines and transplanted tumors with little toxicity to normal host tissues. However, the mechanisms underlying anticancer activity of glucosamine are poorly understood. To study the mechanisms, the human prostate cancer DU145 cells were used for the model.

**Results:**

Glucosamine at concentration 2 mM suppressed proliferation and induced death of DU145 cells. Detailed analysis showed that glucosamine decreased DNA synthesis, arrested cell cycle at G1 phase and induced apoptosis. The effects of glucosamine were associated with up-regulation of p21waf1/cip, a CDK inhibitor. Our further studies identified glucosamine as an inhibitor of signal transducer and activator of transcription (STAT) 3 which is constitutively activated in many cancer cells including DU145 cells. Glucosamine inhibited phosphorylation of STAT3 at the Tyr705 residue and as a result, reduced STAT3 DNA binding and transcriptional activities. Indeed, the expression of apoptosis inhibitor survivin, which is well known target of STAT3, was suppressed. Contrary to DU145 cells, glucosamine did not affect proliferation of other human prostate cancer PC-3 and C4-2B cells, in which STAT 3 signal pathway is not constitutively active.

**Conclusion:**

Our data identifies glucosamine as a suppressor of STAT3 signaling and suggests that anticancer activity of glucosamine may be attributed to the suppression of STAT3 activity. Potential application of glucosamine for the treatment of tumors with constitutively active STAT3 is proposed.

## Background

D-Glucosamine (2-amino-2-deoxy-D-glucose) is a naturally occurring amino monosaccharide and is an essential carbohydrate component of glycoproteins, glycolipids, and glycosaminoglycans. *In vivo*, glucosamine is synthesized in the phosphorylated form glucosamine-6-phosphate (glucosamine-6-P) from fructose-6-phosphate (fructose-6-P) and glutamine by glucosamine: fructose-6-phosphate amidotransferase, which is the first and rate-limiting step of the hexosamine biosynthetic pathway (HBP). In humans, the endogenous production of glucosamine is in ranges from 4 to 20 g/day [1]. Exogenous glucosamine is actively transported into the animal cells by glucose transporters (GLUTs) [2], and phosphorylated to glucosamine-6-P by hexokinase. Glucosamine-6-P is converted either back to fructose-6-P by deamination for glycolysis pathway [3], or to UDP-N-acetylglucosamine which serves as a donor of N-acetylglucosamine (GlcNAc) for O- or N-linked protein glycosylation [4]. Glucosamine is one of the most popular dietary supplements sold in the United States to ease symptoms of pain related to osteoarthritis [5]. Anticancer activity of glucosamine was first demonstrated more than 50 years ago [6]. Glucosamine is toxic to several malignant cell lines *in vitro*, and an effective lytic agent for several types of transplanted tumors *in vivo*, with little toxicity to normal host tissues [7]. It has been shown that glucosamine induces multiple biochemical and cellular effects including depletion of cellular nucleotide pools, inhibition of protein, RNA and DNA synthesis, and alterations of plasma and intracellular membranes [8]. Recent *in vitro *studies have demonstrated that glucosamine induces apoptosis and suppresses proliferation of the SMMC-7721 hepatoma [9] and K562 leukemia cells [10]. However, the molecular mechanisms underlying anticancer activity of glucosamine are still poorly understood.

Signal transducers and activators of transcription (STAT) are the latent transcription factors that mediate cellular responses to cytokines and growth factors [11]. Some members of the STAT protein family, particularly STAT3 and STAT5, regulate several oncogenic processes such as proliferation, survival, angiogenesis and immune response [12], and activated STAT3 pathway is frequently found in different human tumors [12] including prostate cancer [13]. Suppressing STAT3 signaling pathway causes growth inhibition and apoptosis of cancer cells [14-16], therefore STAT3 represents a validated target for cancer therapy [12,17]. Prostate cancer is the most common cancer among men in USA, comprising one-third of all new cancer cases each year, and is the second leading cause of cancer-related death in males (over 30,000 per year) [18]. Despite significant advances in surgical techniques and treatment options including androgen ablation therapy, radiotherapy and chemotherapy [19], the recorded number of prostate cancer deaths in United States has not changed from 1989 to 2002 [18]. At present, there is no effective treatment for hormone-refractory prostate cancer which typically develops after androgen ablation therapy [20]. Thus, continued search for the novel prevention and treatment strategies is inevitable.

The goal of the present study was to investigate mechanisms underlying anti-cancer activity of glucosamine in human prostate cancer cells. We found that glucosamine inhibited DNA synthesis, induced cell-cycle arrest at G1 phase and stimulated apoptosis in the human prostate cancer DU145 cells. Our studies identified glucosamine as a previously unrecognized suppressor of STAT3 signaling pathway. In DU145 cells, STAT3 is constitutively activated and is required for cell proliferation and survival. Glucosamine inactivated STAT3 by suppressing phosphorylation at the Tyr705 residue, thereby inhibiting the DNA binding and transcriptional activities, and suppressing its downstream gene expression survivin, an inhibitor of apoptosis. As expected, glucosamine has almost no effects on proliferation of human prostate cancer PC-3 and C4-2B cells, in which STAT3 is not constitutively active. However, similar to DU145 cells, the proliferation of Hela cells harboring constitutively activated STAT3 is suppressed by glucosamine treatment.

## Results

### Glucosamine inhibits DU145 cells proliferation

**Figure 1:**
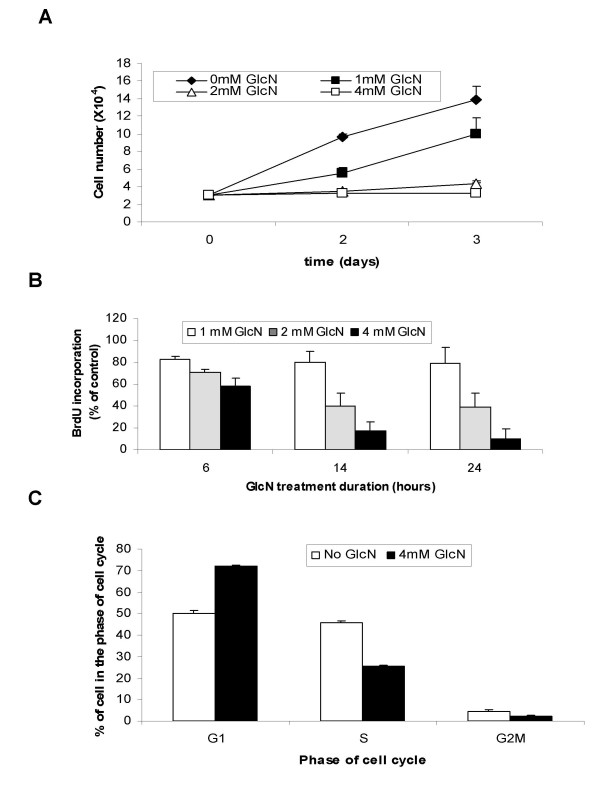
**Glucosamine-induced inhibition of growth of DU145 cells**. **A**, DU145 cells were cultured in the absence or presence of 1, 2, or 4 mM glucosamine for 2 or 3 days in 24-well plates. Total number of cells for each well was counted using a hemocytometer. **B**, BrdU incorporation into DNA of DU145 cells treated with 1, 2, or 4 mM glucosamine for 6, 14, or 24 h is present as % of control without glucosamine. **C**, Flow cytometry analysis of distribution of DU145 cells in different phases of cell cycle after treatment with 4 mM glucosamine for 24 h. The results of the representative experiment are presented as mean ± standard deviation of the three independent samples. All experiments were repeated at least three times.

To examine anti-tumor effects of glucosamine on human prostate cancer cells, the hormone refractory human prostate carcinoma DU145 cells were plated and treated with glucosamine at concentrations 1, 2, and 4 mM for 2 and 3 days. The cells attached to culture flasks were collected and cell numbers were counted using hemocytometer. As shown in Fig. 1A, glucosamine inhibited the proliferation of DU145 cells in a dose-dependent manner. With 1 mM glucosamine, the cell numbers were reduced to 50 and 70% of those of the untreated controls for 2 and 3 days, respectively, and with 2 and 4 mM glucosamine cell proliferation were completely suppressed for 2 and 3 days (Fig. 1A). Since effects of glucosamine on cell proliferation was evident at 2 mM, all subsequent experiments were performed using this concentration unless specified otherwise.

### Glucosamine inhibits DNA synthesis and arrests cell cycle progression from G1 to S in DU145 cells

We analyzed the effects of glucosamine on DNA synthesis by measuring the rate of BrdU incorporation into newly synthesized DNA after culturing cells in the presence of 1, 2 or 4 mM glucosamine for 6, 14, and 24 h. The dose-dependent decrease of BrdU incorporation was observed at 2 mM and 4 mM concentrations examined at all time points, and the inhibition of DNA synthesis became more significant with the duration of the treatment (Fig. 1B). However, glucosamine at 1 mM did not significantly affect DNA synthesis. This data suggests that the decrease of cell numbers at 1 mM concentration (Fig. 1A) is the result of the increase of cell death rather than the inhibition of cell proliferation. Using the same experimental design, we examined whether DNA synthesis in DU145 cells is inhibited by two other hexosamines, galactosamine and mannosamine. In contrast to glucosamine, neither galactosamine nor mannosamine at 2 or 4 mM concentration had any apparent effect on BrdU incorporation (data did not show) indicating the specific role of glucosamine in DNA synthesis inhibition. Furthermore, we analyzed potential effects of glucosamine on cell-cycle progression. DU145 cells were treated in early log-phase with 4 mM glucosamine for 24 h and cell cycle phase distribution was analyzed by flow cytometry. The treatment increased the percentage of cells present in G1 phase from 50% to 73%, and reduced that of S-phase cells from 46% to 25%, and G2M cells from 4% to 2% (Fig. 1C). These results indicate that inhibition of DNA synthesis and cell cycle arrest at G1-S transition play major rolls in the inhibition of cell proliferation by glucosamine.

### Glucosamine induces p21^Waf1/Cip ^expression in DU145 cells

**Figure 2:**
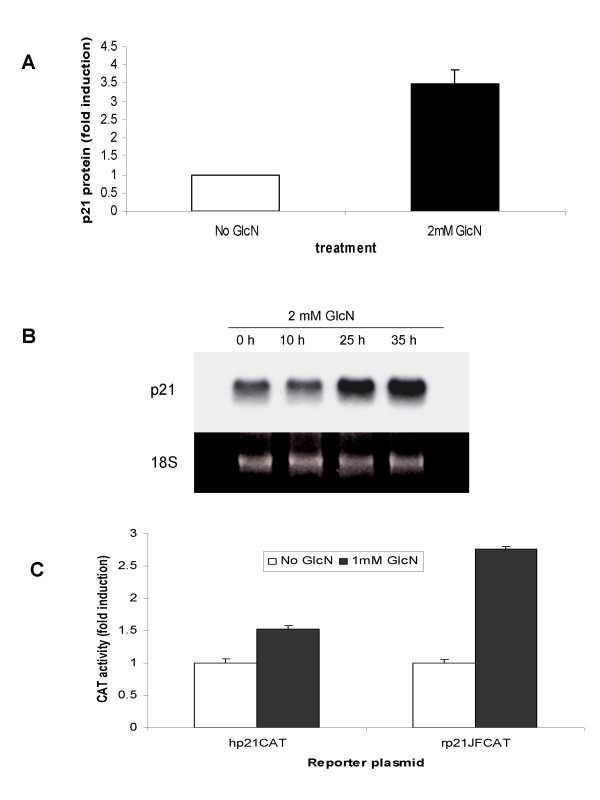
**Induction of the p21/WAF1 expression by glucosamine in DU145 cells**. **A **and **B**, Cells were cultured either without or with 2 mM glucosamine. **A**, p21 protein levels assessed by ELISA 48 h after glucosamine treatment; **B**, p21 mRNA levels were determined by Northern blot 10, 25, and 35 h after glucosamine treatment, representative blot from three independent experiments with similar results is shown. **C**, cells were transfected with human (hp21CAT) and rat (rp21JFCAT) p21 promoter reporter plasmids in the absence or presence of 1 mM glucosamine and processed for CAT activity assay 48 h later. **A **and **C**, the results of a representative experiments were presented as mean ± standard deviation of the three independent samples. All experiments were repeated at least three times.

To examine the mechanisms of glucosamine-induced cell cycle arrest, we analyzed the expression of cyclin-dependent kinase (CDK) inhibitors p21^Waf1/Cip ^and p27^Kip1^, proteins that could arrest cell cycle progression at both G1 and G2 phases [21]. Treatment of DU145 cells with 2 mM glucosamine for 48 h significantly increased the p21 protein (Fig. 2A). To study the regulation of p21 expression, we measured the steady state level of the p21 mRNA in DU145 cells. Northern blot analysis of the total RNA from the treated DU145 cells showed that glucosamine increased the p21 mRNA in a time-dependent manner (Fig. 2B). In contrast, p27 mRNA levels were decreased by this treatment (data not shown.

To explore the possibility that glucosamine affects the p21 promoter activity, we transiently transfected DU145 cells with p21 promoter reporter plasmids in the presence or absence of glucosamine. We found that 1 mM glucosamine induced both the human and rat p21 promoter activities in the cells (Fig. 2C) indicating that the p21 gene is a transcriptional target of glucosamine. These results suggest that the up-regulation of p21 underlies glucosamine-induced inhibition of G1-S transition (Fig. 1C).

### Glucosamine induces cell death in DU145 cells

**Figure 3:**
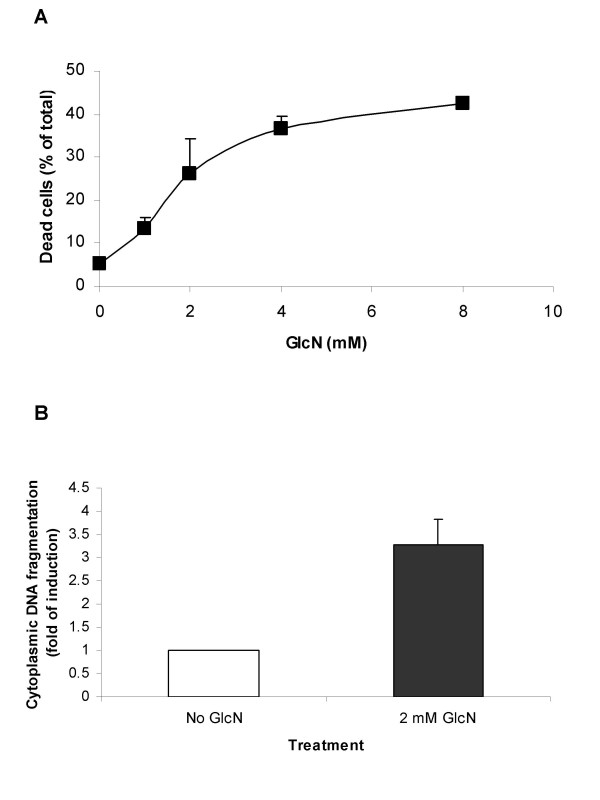
**Induction of DU145 cell death by glucosamine**. **A**, DU145 cells were cultured in the absence or presence of 1, 2, 4 or 8 mM glucosamine for 2 days in 24-well plates. Percent of dead cells was examined by trypan blue staining. **B**, apoptosis in DU145 cells after treatment with 2 mM glucosamine for 24 h. The results of a representative experiment are presented as mean ± standard deviation of the three independent samples. All experiments were repeated at least three times.

We estimated percentages of dead cells by trypan blue staining after treatment of DU145 cells with four different concentrations 1, 2, 4, or 8 mM of glucosamine for two days. As shown in Fig. 3A, glucosamine induced cell death in a dose-dependent manner and a significant percentage of dead cells were detected even at 1 mM concentration. It notes that the effect on cell death reached to plateau with approximately 40% of dead cells at 8 mM concentration. To study the nature of cell death, apoptosis assays were carried out. Treatment with 2 mM glucosamine for 24 h resulted in 3-fold increase in DNA fragmentations as detected by ELISA apoptotic cell death assay (Fig. 3B). Glucosamine-induced apoptosis was further confirmed by flow cytometry analysis after annexin V staining of glucosamine-treated cell culture (data not shown). Taken together, our results show that glucosamine exerts anticancer activities in DU145 cells through at least two pathways by inhibiting cell proliferation and stimulating cell death.

### Glucosamine inhibits STAT3 signaling pathway in DU145 cells

**Figure 4:**
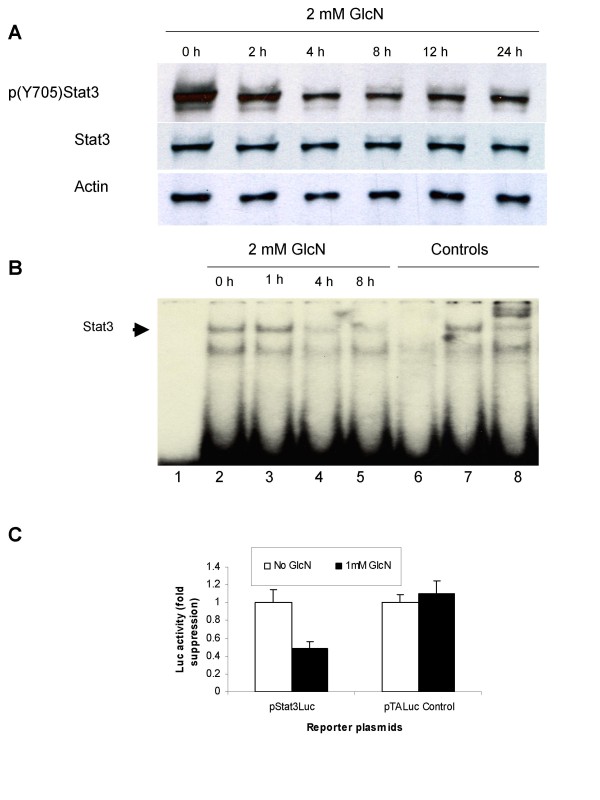
**Glucosamine inhibits STAT3 activity in DU145 cells**. **A**, Control and glucosamine-treated cells were grown in 6-well plates either without or with 2 mM glucosamine and whole-cell extracts were analyzed by Western blot 2, 4, 8 and 24 h after glucosamine treatment. **B**, EMSA. Lane 1, free probe without nuclear proteins. Lanes 2-5, binding of the STAT3 radioactive probe to nuclear proteins from DU145 cells, untreated (lane 2) or treated with 2 mM glucosamine for 1 h (lane 3), 4 h (lane 4) and 8 h (lane 5). Lane 6-7, competition assay with 100-fold molar excess of non-radioactive STAT3-specific competitor (lane 6) or nonspecific competitor (lane 7). Lane 8, nuclear proteins from untreated DU145 cells incubated with STAT3-specific antibody before incubation with radioactive probe. Representative data from three independent experiments with similar results is shown. **C**, cells were transfected with pSTAT3-Luc luciferase reporter plasmid under the control of STAT3 responsible element, or control pTALuc plasmid, and were cultured without or with 1 mM glucosamine for 48 hours before luciferase activities were measured in cell extracts. The results are presented as mean of three independent experiments ± standard deviation.

Proliferation of DU145 cells *in vitro *is stimulated by interleukin-6 (IL-6) in an autocrine manner [22] and is associated with constitutive activation of STAT3 signaling pathway [14]. The blockage of STAT3 in these cells by either antisense oligonucleotides or dominant negative STAT3 proteins was shown to inhibit cell proliferation and induce apoptosis [14]. These data led us to examine whether glucosamine affects STAT3 pathway. When activated, STAT3 is phosphorylated, and the DNA binding and transcriptional activities are enhanced to stimulate cell proliferation and survival. We assessed phosphorylation of STAT3 at the Tyr705 residue by Western blot analysis, DNA binding activity by EMSA, and transcriptional activity by transient transfection assays. For the phosphorylation, DU145 cells were treated with 2 mM glucosamine for 2, 4, 8, 12 or 24 h and the whole cell lysates were analyzed by Western blotting using antibodies specific to phosphorylated and nonphosphorylated STAT3 proteins. The results showed that glucosamine treatment gradually decreased the phosphorylation at the Tyr705 residue for 8 hours, and the lower phosphorylation levels were sustained for up to 24 h (Fig. 4A). Dose-dependent experiments revealed a clear decrease of STAT3 phosphorylation at Tyr705, but phosphorylated STAT3 did not disappear completely even under 8 mM concentration (Additional file 1). On the contrary, the treatment did not affect the quantity of total STAT3 proteins for at least 24 h; therefore, the diminished phosphorylation at the Tyr705 residue is not the consequence of the down-regulation of STAT3 proteins.

To examine *in vitro *STAT3 DNA binding activity, EMSA was performed with a mixture of a radioactive oligodeoxyribonucleotide probe specific to STAT3 and nuclear extracts from DU145 cells treated with 2 mM glucosamine for 1, 4, and 8 h (Fig. 4B). A complex formation between STAT3 proteins and the probe indicated by an arrowhead (lane 2), disappeared in the presence of an excess of the non-radioactive probe (lane 6), but not with an excess of the non-specific oligodeoxyribonucleotide (lane 7). The complex was super-shifted with the specific anti-STAT3 antibody (lane 8). This data demonstrated that the complex formation is specific between STAT proteins and the probe. The formation was reduced with glucosamine treatment in a time-dependent manner; almost no changes were observed 1 h after the treatment (lane3), and the complex formation was considerably reduced 4 h (lane 4) and remained lower 8 h (lane 5) after the treatment. It seems that the reduction in phosphorylated STAT3 (Fig. 4A) is consistent with that in the DNA binding activity (Fig. 4B).

To study the effects of glucosamine on the STAT3 transcriptional activity, we carried out transient transfection assays using the reporter plasmid pSTAT3-Luc in which the luciferase gene expression was under the control of two STAT3 response elements. The plasmid pTA-Luc lacking the STAT3 response element was used as a control plasmid. After transfection with pSTAT3-Luc or pTA-Luc, DU145 cells were treated with 1 mM glucosamine for 48 h and luciferase activity was measured (Fig. 4C). Glucosamine treatment reduced the luciferase activity of pSTAT3-Luc to 50% of that without the treatment, while the treatment did not affect the luciferase activity in the cells transfected with the control plasmid. These data demonstrates that glucosamine suppresses the transcriptional activity of STAT3 and can be an effective inhibitor of STAT3 signal pathway in DU145 cells.

### Glucosamine inhibits survivin gene expression in DU145 cells

**Figure 5:**
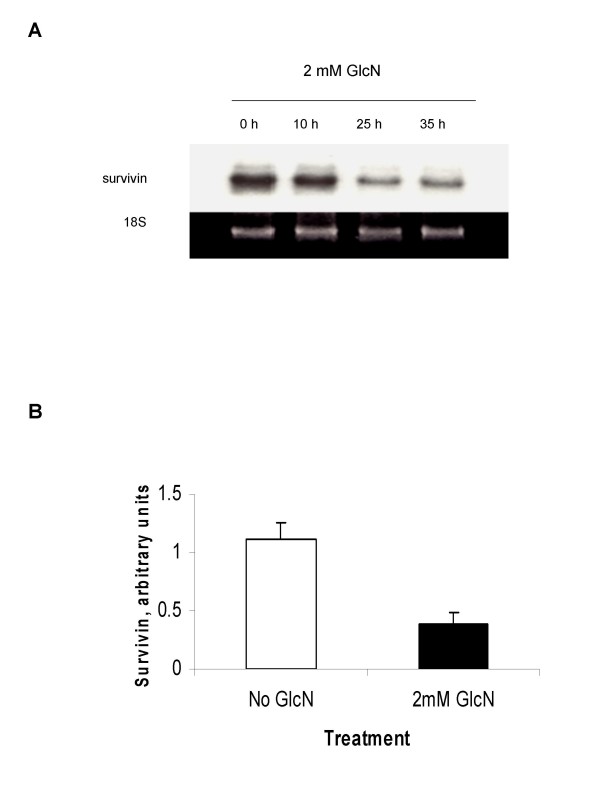
**Suppression of survivin expression in DU145 cells by glucosamine**. **A **and **B**, cells were cultured either without or with 2 mM glucosamine. **A**, survivin mRNA levels were analyzed by Northern blot 10, 25, and 35 h after glucosamine treatment. The representative blot of three independent experiments is shown: **B**, survivin protein level were analyzed 35 h after glucosamine treatment by human total Survivin enzyme immunometric assay kit. The results are presented as mean ± standard deviation of three independent experiments.

Since an apoptosis inhibitor survivin is a transcriptional target of STAT3 and one of validated cancer therapeutic target [23], we investigated whether the expression of survivin in DU145 cells was affected by glucosamine. The total RNAs from the cells treated with 2 mM glucosamine were isolated and analyzed by Northern blot as shown in Fig. 5A. Survivin mRNA levels did not change for the first 10 hours, but were considerably decreased for 25 h and remained lower levels for up to 35 h after glucosamine addition. Survivin protein in total cell lysates was also measured by enzyme immunometric assays. The treatment of DU145 cells with 2 mM glucosamine for 35 h reduced the amount of survivin protein to one-third of the levels observed in non-treated cells (Fig. 5B). These findings show that glucosamine is a suppressor of the survivin gene expression. This effect likely occurs through suppression of STAT3 signaling and may contribute to cell proliferation restraint and apoptosis observed in glucosamine-treated cells.

### Specificity of glucosamine-induced inhibition of cancer cell proliferation

**Figure 6:**
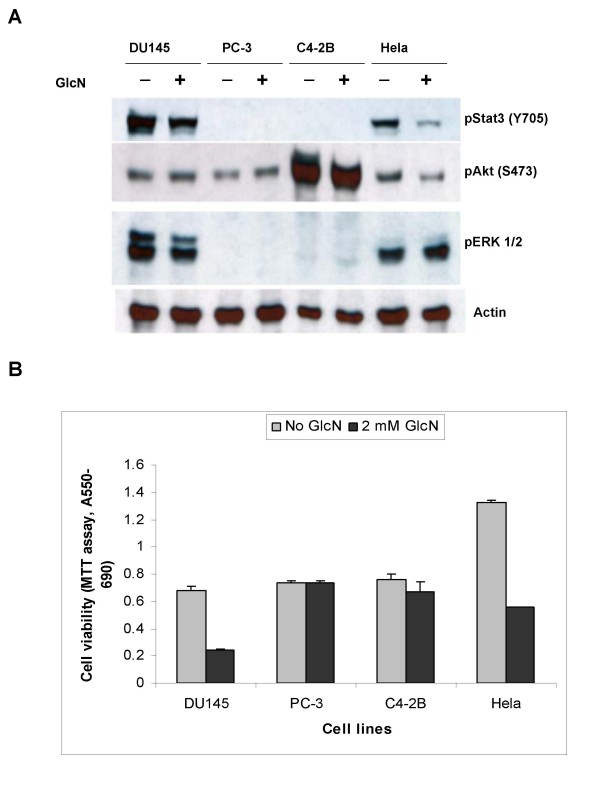
**Inhibition of cancer cells growth by glucosamine is associated with the suppression of STAT3 (Y705) phosphorylation**. **A**, DU145, PC-3, C4-2B and Hela cells grown in 6-well plates either left untreated or were treated with 2 mM glucosamine for 24 h, and whole-cell extracts were used for Western blot analysis. Representative blot from at least three independent experiments with similar results is shown. **B**, Viability of cells grown in 96-well plates without or with 2 mM glucosamine for 3 days. Viability was assessed by MTT assay. All experiments were repeated at least three times with similar results. The results of a representative experiment are presented as mean ± standard deviation of three independent samples.

To examine whether glucosamine effects on the suppression of cell proliferation is specific to STAT3 pathway in DU145 cells, we investigated effects of glucosamine in three additional cancer cell lines, prostate carcinoma PC-3 and C4-2B cells and cervix carcinoma Hela cells, in which different signaling pathways are activated. In addition to STAT3 pathway, well studied cell-signaling pathways associated with cell proliferation and survival, such as phosphatidylinositol 3-kinase (PI3K)/Akt and mitogen-activated protein kinases (MAPK), were investigated. We compared the phosphorylation levels of STAT3 (Tyr-705), Akt (Ser-473), and Extracellular-Signal-Regulated Kinase ERK1/2 (p44/42 MAPK) (Thr202/Tyr204), since the phosphorylation of these proteins is co-related with the activation of corresponding pathways. These cancer cells were treated with 2 mM glucosamine for 18 h and whole-cell extracts were used for Western blot analysis. As shown in Fig. 6A, in DU145 and Hela cells all three signaling pathways were constitutively activated, but the treatment mainly suppressed the STAT3 phosphorylation. In contrast to these cells, in PC-3 and C4-2B cells only PI3K/Akt pathway was constitutively active and the treatment did not change phosphorylation levels of Akt (Ser473). It concludes, therefore, that glucosamine specifically inactivates STAT3 pathway, but has almost no effects on PI3K/Akt and ERK pathways under the conditions. This conclusion is consistent to the analysis of cell proliferation (Fig. 6B) showing that glucosamine is an effective inhibitor of proliferation only in cells where STAT3 pathway is active, but ineffective in cells where the pathway is not active.

## Discussion

Although anticancer activity of glucosamine was shown more than 50 years ago [6], molecular mechanisms of its actions remained unclear [8,10]. By testing human prostate cancer cell lines, we have found that glucosamine dose-dependently suppresses proliferation and increases apoptosis, and these anti-proliferative effects depend on the status of STAT3 pathway in these cells. We show here that glucosamine specifically inactivates STAT3 signaling pathway and restrains the proliferation of cells expressing constitutively activated STAT3, but is ineffective in cells that do not have the activated pathway.

STAT3 is a transcription factor that stimulates cell proliferation and survival, and frequently exhibits constitutive activity in different types of human tumors including prostate, lung and breast cancers. Intervention of activated STAT3 is a promising therapy to treat malignant cancers [12,17]. STAT3 normally resides in the cytoplasm. After phosphorylation of the Tyr705 residue, STAT3 dimerizes and translocates to the nucleus where it binds to the specific DNA sequences to control transcription of several cell cycle- and apoptosis-regulatory genes [11]. Blocking the pathway by either antisense oligonucleotides or dominant-negative proteins induced growth retardation and apoptosis in DU145 cells [14,24]. In this study we demonstrate that glucosamine suppresses the phosphorylation of STAT3 (Tyr705), thereby inhibiting its DNA binding and transcriptional activities, and retards the proliferation of DU145 and Hela cells (Fig. 6B) and the leukemia K562 cells (our unpublished data and ref. 10), all of which express constitutively active phosphorylated STAT3. However, glucosamine did not affect the proliferation of PC-3 and C4-2B cells, in which STAT3 pathway is not active (Fig. 6A). Our findings are in good agreement with the recent publication reporting that glucosamine inhibited proliferation, and restrained cell cycle G1-S phase transition of DU145 cells, but did not have any major effects in PC-3 cells [25].

We demonstrate that expression of genes controlling cell-cycle and apoptosis such as p21, p27 and survivin is affected by glucosamine treatment. Glucosamine-induced arrest of DU145 cells at G1 phase could be partially due to up-regulation of p21 through the inactivation of STAT3 pathway. Indeed, it was reported that over-expression of p21 in DU145 cells blocked G1-S progression [26] and that inactivation of JAK3/STAT pathway in cancer cells increased p21 expression [27]. Thus, it is likely that observed induction of p21 by glucosamine in DU145 cells is a consequence of glucosamine-induced STAT3 inactivation. In contrast to the p21 expression, glucosamine reduced p27 mRNA levels in DU145 cells (our unpublished results). Similar p21 and p27 expression patterns were reported by others in renal mesangial cells treated with glucosamine [28]. Reduced expression of survivin should contribute to the increase of apoptosis by glucosamine. Since survivin gene expression is under control of STAT3, it is reasonable to conclude that the expression is down-regulated by inactivation of STAT3 signaling with glucosamine treatment. Taken together, the present study demonstrates that anticancer action of glucosamine is at least in part due to the blockage of STAT3 signaling pathway. Glucosamine may not be a very potent STAT3 inhibitor; fig. 3A showed less than 50% cell death after treatment with 8 mM glucosamine and fig. 4A revealed the presence of residual phosphorylated STAT3 after treatment with 2 mM glucosamine for 24 h. These results suggest that there could be more than two pathways to activate STAT3 in DU145 cells, one sensitive and the other not sensitive to glucosamine treatment (further discussed below). It might be possible that this residual active STAT3 could support survival of the remaining cells and therefore, more inhibition of STAT3 phosphorylation would be required for death of more tumor cells. However, glucosamine may have an application for the treatment of tumors with constitutively active STAT3, because it has several unique features as a putative therapeutic agent. First, glucosamine has a low toxicity as proven for many years by taking to ease symptom of pain related to osteoarthritis [5]. Second, preferential uptake by tumor cells is expected, since glucosamine is transported into cells through glucose transporters that are much more active in cancer cells than normal cells [29]. Third, glucosamine enhances the potency of anticancer agents in cancer cells [[30], and our unpublished results]. Taken into account of all, we believe that glucosamine warrants further study as a therapeutic agent.

Molecular mechanisms underlying the inhibition of STAT3 activity by glucosamine remain to be determined. STAT3 is phosphorylated on the tyrosine 705 residue by three types of kinases: receptor tyrosine kinases such as EGFR, FGFR and platelet-derived growth factor receptor (PDGFR), Janus kinase (JAK) family members which constitutively bind to cytokine receptors, or cytoplasmic kinases including Src and Abl [12]. Protein tyrosine phosphatases such as the SH2-domain containing family can directly dephosphorylate active STAT3. Glucosamine might deactivate or activate these kinases or phosphatases, respectively to de-phosphorylate STAT3 proteins. It has been shown that glucosamine increases O-glycosylation of nuclear and cytosolic proteins which modifies their functions [4,31]. It is therefore plausible that proteins involved in these kinase and phosphatase reactions could be modified by O-glycosylation and affect indirectly STAT3 signaling. Alternatively, STAT3 itself is modified by O-glycosylation in HC11 mammary epithelial cells by EGF [32] and in DU145 cells by glucosamine (our unpublished results), but functional consequences of this modification are to be determined. Additionally, glucosamine could suppress the protein functions through inhibition of protein N-glycosylation as reported for influenza virus hemagglutinin [33] and COX-2 [34]. Since many receptors including growth factor and cytokine receptors are N-glycosylated, it is conceivable that glucosamine could inactivate their functions by inhibition of N-glycosylation thereby suppressing STAT3 signaling.

## Conclusion

Studies on the effects of glucosamine on human prostate carcinoma DU145 cells *in vitro *identified several molecular events in its anti-tumor activity: up-regulation of CDK inhibitor p21waf1/cip expression, down-regulation of apoptosis inhibitor survivin and the most important suppression of STAT3 signaling pathway. STAT3 is activated in many different cancers including colon, breast and prostate cancers. The activation often is associated with transition from hormone-sensitive to hormone-refractory prostate cancer and promotes its metastatic progression. In addition, activated STAT3 stimulates survival and proliferation of tumors. Although more work is required to fully understand mechanisms of anticancer action of glucosamine, this study provides the basis for the potential application of glucosamine as an inhibitor of STAT3 signaling pathway in cancer cells.

## Methods

### Cell lines and reagents

The human prostate cancer DU145 and PC3 and cervical cancer HeLa cell lines were obtained from the American Type Culture Collection. The human prostate cancer C4-2B cell line was purchased from ViroMed Laboratories (Minnetonka, MN). Cells were cultured in Eagle's minimum essential medium supplemented with glutamine, essential amino acids, 10% fetal bovine serum and antibiotics (100 units/ml penicillin G and 100 units/ml streptomycin sulfates). Cells were incubated at 37°C in 5% CO2, and the medium was changed every 3-4 days. Cells were passaged at 70% confluent using trypsin/EDTA. D-Glucosamine hydrochloride and trypan blue solution were purchased from Sigma Chemical Co. (St. Louis, MO). Specific phosphatidylinositol 3-kinase inhibitor LY294002 and inhibitor of MAP kinase kinase (MEK) PD98059 were purchased from Calbiochem (La Jolla, CA).

### Cell growth and cell death assays

Cells in exponential growth were harvested, plated at a density of 3 × 10^4 ^cells per well in 24-well flat-bottomed plates (Corning, Inc., Corning, NY) for 24 h, fed with fresh medium and treated with different concentrations of glucosamine. After 2 and 3 days, both floating and attached cells were harvested by trypsinization and collected by centrifugation. Cell pellets were resuspended in fresh media, and trypan blue solution was added at a ratio of 1:1. The total and trypan blue-positive (blue) cells for each well were counted using a hemocytometer. The total cells were expressed as cell number per well. The blue cells were considered dead and were counted as a percentage of 300 the total cells. Cell growth was also examined by colorimetric assay of cell proliferation with cell proliferation kit I (MTT) (Roche Applied Science, Indianapolis, IN) according to the manufacturer's protocol. Cells were plated in triplicate at a density of 2.5 × 10^3 ^cells/well in 100 ul culture medium containing various tested compounds into 96 well flat bottom microplates and the effects of compounds on cell growth were measured by 1, 2 or 3 days after plating. Data was presented as mean ± standard deviation (SD) of the three wells. Each cell line and each compound was analyzed in three independent experiments.

### DNA synthesis assay

DNA synthesis was determined by measurement of bromodeoxyuridine (BrdU) incorporation into DNA via a nonradioactive colorimetric assay using ELISA (Roche Applied Science, Indianapolis, IN). Cells in exponential growth were harvested and plated at a density of 3 × 10^3 ^cells per well in 96-well flat-bottomed plates (Corning, Inc., Corning, NY) for 24 h. Cells were fed with fresh medium and then treated with 1, 2 or 4 mM hexosamines. The amount of incorporated BrdU over a 2 h period was measured at 6, 14 and 24 h after the hexosamine treatment. The assay was carried out according to the manufacturer's protocol. The substrate reaction was measured without a stop solution at 370 nm on a spectrophotometer. For each treatment and time point three wells were used. The data was presented as mean and standard deviation (SD) of the three independent wells.

### Cell cycle analysis

Cells were plated at a density of 1.5 × 10^4 ^cells per cm^2 ^in a 250 ml tissue culture flask (BD Bioscience, MA). After 24 h, cells were fed with fresh medium and treated with 4 mM GlcN. Both adherent and detached cells were collected by trypsinization, washed in PBS and fixed in ice-cold 80% ethanol for at least 2 h. Fixed cells were centrifuged at 400 × g and resuspended in propidium iodide (PI) stain buffer (0.3% Nonidet NP-40, 0.5 mg/ml of DNase-free RNase A and 0.05 mg/ml of PI in PBS) for 30 minutes. After staining, the samples were analyzed by a flow cytometer with the fluorescence-activated cell sorter.

### Quantification of apoptosis

Apoptosis in DU145 cells was quantified by detection of mono- and oligonucleosomes in the cytoplasm of cells by photometric enzyme immunoassay with the Cell Death Detection ELISA plus kit (Roche Applied Science, Indianapolis, IN) according to the manufacturer's protocol. Cells were cultured in a 24 well plate for 24 h, and then treated with 2 mM GlcN for 24 h. Both floating and adherent cells were used for assays. The samples were analyzed in duplicate in three independent experiments. Results were measured by absorbance at 405/490 nm and expressed as a fold of induction of DNA fragmentation relative to the control without GlcN treatment.

### Protein immunoassays

The quantification of p21/WAF1 protein in DU145 cells was performed by the p21WAF1 ELISA kit (EMD Biosciences, Inc, San Diego, CA, Cat. No. QIA18) according to the manufacturer's protocol. The samples were analyzed in duplicate in three independent experiments. Optical absorbance was measured at 450-550 nm and expressed as p21 amount relative to controls without GlcN treatment. For the quantitative determination of survivin, the human Total Survivin Enzyme Immunometric assay kit (Assay designs Inc, Ann Arbor, MI, Cat. No. 900-111) was used. The cells were lysed directly in the wells of 6-well plates and analysis of lysates was performed according to the manufacturer's protocol. The samples were analyzed in duplicate in three independent experiments and optical absorbance was measured at 450 nm and expressed as survivin arbitrary units.

### RNA extraction and Northern blotting

Cells were lysed in a culture dish with TRIzol reagent (Invitrogen Corporation Carlsbad, CA) by using 2 ml per 50-75 cm^2 ^and RNA was isolated according to the manufacturer's recommendations. For Northern blot analysis, 15 μg of total RNA were electrophoresed on 1% agarose-formaldehyde gels, transferred to Nylon filters (Zeta-Probe GT; Bio-Rad Laboratories, Hercules, CA), ultraviolet cross-linked (Stratagene, La Jolla, CA), and hybridized with a P^32^-labeled single strand DNA probe in the ULTRAhyb hybridization buffer (Ambion, Austin, TX).

The probes were made by PCR reaction using cDNAs template, one sequence-specific primer, dNTPs and P^32^-labeled α-dNTP (mainly dCTP). After washing, filters were exposed for autoradiography at -70°C with a BioMax screen (Eastman Kodak, Rochester, NY).

### Transient transfection analysis

The human CAT reporter plasmid was constructed by inserting a 2.7 kB PCR fragment of the human p21 promoter region (-2583/+110) in the pCAT Basic plasmid (Promega Corporation, Madison, WI). The rat CAT reporter plasmid (p21JFCAT) with a 4.7 kB rat p21 promoter region in the pJFCAT plasmid was a gift from Dr. Bert Vogelstein. STAT3 transcriptional activity was examined by transient transfection assays of the pSTAT3-Luc reporter plasmid. As a control, the pTA-Luc plasmid which does not carry STAT3 responsible DNA elements was used. Both the pSTAT3-Luc and pTA-Luc plasmids were purchased from Panomics Inc. (Fremont, CA, Cat. No. LR0077 and LR0000). For transfection, DU145 cells were plated at density of 2 × 10^5^cells per well in 6-well flat-bottomed plates (Corning, Inc, NY) for 24 h. One hour before transfection, the cells were fed with fresh medium with 1 mM GlcN. Transfections were performed in triplicate using the siPORT XP-1 transfection agent (Ambion Inc., Austin) with 0.7 μg of the reporter or control plasmid and 0.3 μg of the β-Gal reporter plasmid. The cells were harvested in a Reporter lysis buffer (Promega Corporation, Madison, WI) 48 h after the transfection and used for CAT, Luciferase and β-Gal activity assays. All transfection were analyzed in three independent experiments and results were expressed as a fold of reporter gene activation or suppression relative to the controls without GlcN treatment.

### Immunoblotting

Control and glucosamine-treated cells were grown in 6-well plates. After removing the culture medium, cells were washed with 1 × PBS and then lysed in the wells with 0.2 ml of RIPA lysis buffer (25 mM Tris:HCl, 150 mM NaCl, 1% NP-40, 1% Sodium deoxycholate, 0.1% SDS, pH 7.6) supplemented with protease and phosphatase inhibitors (Santa Cruz Biotechnology, Santa Cruz, CA) for 15 min at 4°C. Lysates were transferred to 1.5 ml microcentrifuge tubes, vortexed at maximum speed for 15 sec to shear DNA and centrifuged at 12000 g for 10 min at 4°C. Supernatants were quantified for protein concentrations by BCA protein assay kit (Pierce Biotechnology, Rockford, IL). Immunoblotting was performed after SDS-PAGE of equal amounts of proteins on 10% precast gels (Pierce, Rockford, IL, Prod # 25204) and were detected using horseradish peroxidase-conjugated antibody and Western blotting luminol reagent (Santa Cruz Biotechnology, Santa Cruz, CA). Antibodies to STAT3, phospho-STAT3 proteins, phospho-Akt (Ser 473) phospho-p44/42 MAPK/ERK1/2 and actin were purchased from Cell Signaling Technology, Inc, (Beverly, MA).

### EMSA

Nuclear extracts were prepared by lysing nuclei in a high-salt buffer supplemented with protease and phosphatase inhibitors (Nuclear extraction kit, Panomics, Fremont, CA, and Cat. No. AY2002) according to the manufacturer's protocol. Protein concentrations were quantified by the Bio-Rad protein assay (Bio-Rad Laboratories, Hercules, CA). Nuclear extracts (10 μg) were incubated in a final volume of 20 μl of 10 mM HEPES (pH 7.5), 50 mM KCl, 10% glycerol, 4 mM spermidine, 100 μg/ml polydI-dC and the P^32^-labeled double-strand oligodeoxyribonucleotide with a STAT3 binding motif AGCTTCATTTCCCGTAAATCCCTA (36) for 20 min at room temperature. For the supershift analysis, nuclear extracts were preincubated with the anti-STAT3 antibody (Chemicon International, Temecula, CA) for 20 min at room temperature. DNA-protein complexes were electrophoresed on 4.5% nondenaturing polyacrylomide gels with 0.5 × TBE buffer containing 2.5% glycerol at room temperature and autoradiophographed.

## List of abbreviations

STAT: signal transducer(s) and activator(s) of transcription; glucosamine-6-P: glucosamine-6-phosphate; fructose-6-P: fructose-6-phosphate; GLUTs: glucose transporters; BrdU: bromodeoxyuridine; SD: standard deviation; PI: propidium iodide; CAT: chloramphenicolacetyltransferase; PCR: polymerase chain reaction; β-Gal: β-galactosidase; EMSA: electrophoretic mobility shift assay.

## Competing interests

The authors do not have any financial or personal relationships with other people or organizations that could inappropriately influence the work described in this manuscript.

## Authors' contributions

VC conceived of the study, designed and performed all of the described experiments with the exception of study effects of glucosamine in PC-3 and Hela cells, and drafted the manuscript. CS carried out the proliferation and western blotting analysis of PC-3 and Hela cells. KI coordinated the study and edited the manuscript. All authors have read and approved the final manuscript.

## Supplementary Material

Additional file 1 **Glucosamine induces dose-dependent inhibition of STAT3 phosphorylation in DU145 cells**. Control and glucosamine-treated DU145 cells were grown in 6-well plates either without or with different concentrations glucosamine (mM) and whole-cell extracts were analyzed by Western blot 8 h after glucosamine treatment. Representative data from three independent experiments with similar results is shown. Click here for file
