# Supplementary figures and images for: Glucosamine suppresses proliferation of human prostate carcinoma DU145 cells through inhibition of STAT3 signaling
Source: Cancer Cell Int. 2009 Sep 10;9:25. doi: 10.1186/1475-2867-9-25 (PMC2747838; doi:10.1186/1475-2867-9-25)

## Slide 1
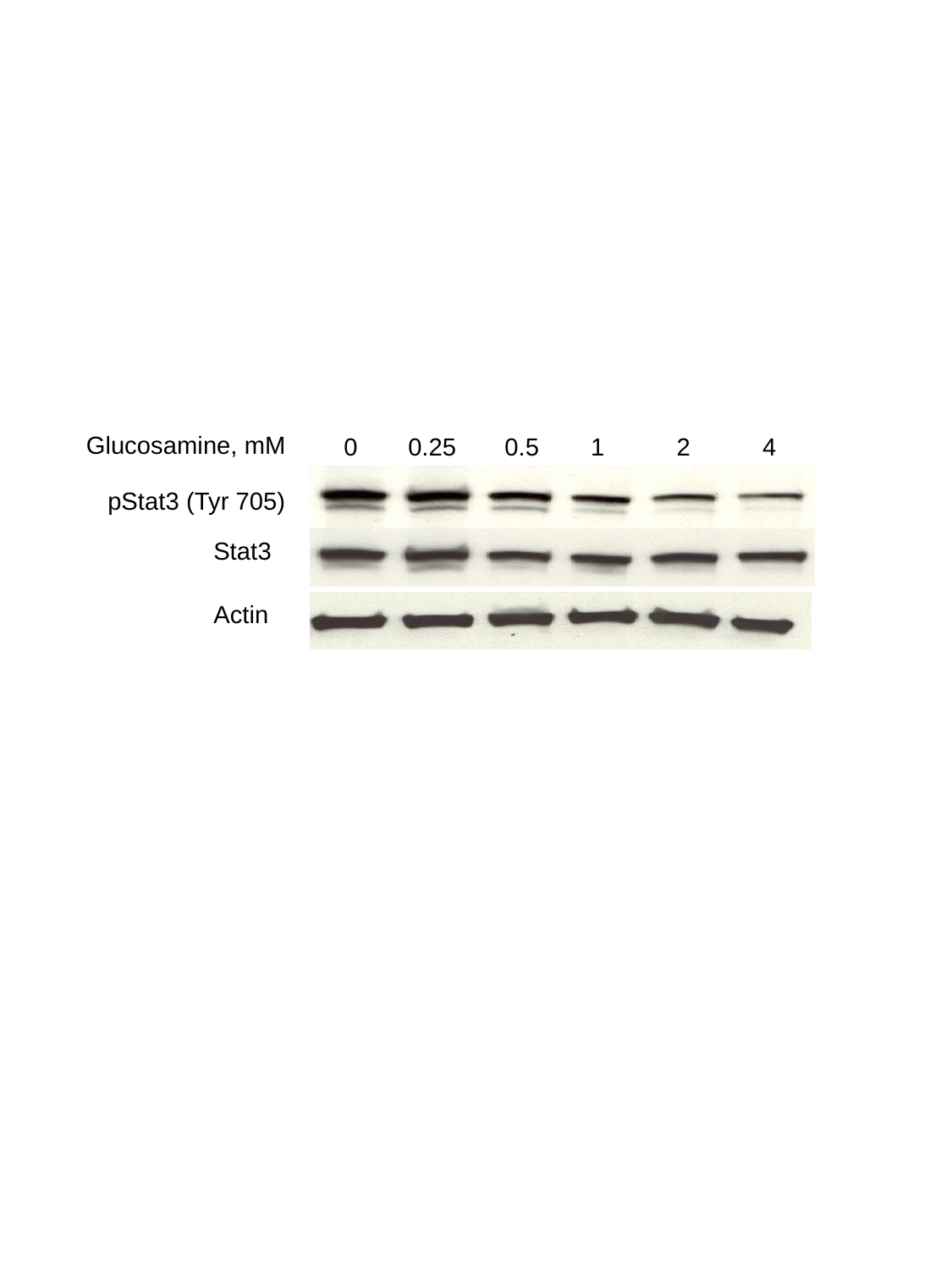

Glucosamine, mM
0
0.25
0.5
1
2
4
pStat3 (Tyr 705)
Stat3
Actin

Supplement: Additional file 1 — Glucosamine induces dose-dependent inhibition of STAT3 phosphorylation in DU145 cells. Control and glucosamine-treated DU145 cells were grown in 6-well plates either without or with different concentrations glucosamine (mM) and whole-cell extracts were analyzed by Western blot 8 h after glucosamine treatment. Representative data from three independent experiments with similar results is shown. [file 1475-2867-9-25-S1.ppt]
